# Palmar and dorsal fixed-angle plates in AO C-type fractures of the distal radius: is there an advantage of palmar plates in the long term?

**DOI:** 10.1186/1749-799X-7-8

**Published:** 2012-02-17

**Authors:** Michael G Jakubietz, Joerg G Gruenert, Rafael G Jakubietz

**Affiliations:** 1 Department of Trauma-, Hand-, Plastic and Reconstructive Surgery, University of Wuerzburg, Wuerzburg, Germany; 2 Department of Hand, Plastic and Reconstructive Surgery Kantonspital, St. Gallen, Switzerland

## Abstract

**Background:**

Current surgical approaches to the distal radius include dorsal and palmar plate fixation. While palmar plates have gained widespread popularity, few reports have provided data on long term clinical outcomes in comparison. This paper reports the result of a randomised clinical study comparing dorsal Pi plates and palmar, angle-stable plates for treatment of comminuted, intraarticular fractures of the distal radius over the course of twelve months.

**Methods:**

42 patients with unilateral, intraarticular fractures of the distal radius were included and randomised to 2 groups, 22 were treated with a palmar plate, 20 received a dorsal Pi-plate. Results were evaluated after 6 weeks, 3, 6 and 12 months postoperatively focussing on functional recovery as well as radiological results.

**Results:**

The palmar plate group demonstrated significantly better results regarding range of motion and grip strength over the course of 12 months. While a comparable increase in function was observed in both groups, the better results from the early postoperative period in the palmar plate group prevailed over the whole course. Radiological results showed a significantly increased palmar tilt and carpal sag in dorsal plates, with other radiological parameters being comparable. Pain levels were decreased in dorsal plates after hardware removal and failed to show significant differences after 12 months. However, complications such as tendon ruptures were more frequent in the dorsal plate group.

**Conclusions:**

Functional advantage of palmar plates gained within the first 6 weeks prevails over the course of a year. Both groups demonstrate further gradual increase of function after 6 months, although dorsal plates did not catch up completely. Improved early postoperative function seems to be the cornerstone for the best possible results. Patients with dorsal plates benefit from hardware removal more than palmar plates in terms of reduction of pain levels. The advantage of palmar plates is a faster functional recovery with lower complication rates. This is especially important in the elderly population. Radiological results did not show a superiority of palmar plates over dorsal plates.

## Introduction

Fractures of the distal radius are the most common fractures in the upper extremity and treatment options have been controversially discussed throughout the literature over the last decades [1-5]. Especially the invention of angle stable palmar plating systems has had a considerable impact by emerging as the currently perceived gold standard. As shown in biomechanical studies, palmar plates allow rigid fixation of cancellous, fragmented bone. A short term follow up study of our work group has also shown striking advantages of palmar over dorsal plates regarding the rapid regain of function in palmar plates [5]. Angle stable palmar plates are now considered to be safe, effective and more physiological [1,3,6,7] The shift to palmar plates is largely unexamined by randomized research, as noted by Martineau [8]. As a reaction some authors have demanded evidence that justifies the change in the management of radius fractures [9]. While a previous report on some of our patients has shown better functional results and considerably less complications in the short term, which in itself justifies the use of palmar plates, few scientific reports about longer term follow up exist [5]. Some surgeons have reported the method of treatment to be of minor importance in the long term, as the fracture pattern seems to predetermine the long term outcome. In an attempt to determine whether the short term advantage of palmar plates prevails in the long term, a larger group of patients was evaluated as a continuation of our previous study [5]. This report, based on an increased number of patients compared to the previously published short term follow up, summarizes the results conducted in St. Gallen. The main objective was not only to evaluate if radiological and functional results displayed a statistical significance and to possibly prove further advantages of palmar plating systems in the longer term, but also to evaluate potential benefits of hardware removal in regard to function, pain, and patient satisfaction.

## Methods

50 patients, operated on in a period of 8 months were initially registered in the study and 42 patients with unilateral AO-type C1, C2 and C3 fractures of the distal radius were included as they completed the complete follow up including hardware removal after 6 months. Patients were randomised to two groups: open reduction and internal fixation with a palmar, angle-stable plate (Aptus Radius, Medartis^®^, Basel, Switzerland) or open reduction and internal fixation with the dorsal Pi-plate (AO-ASIF Pi-Plate, Synthes^®^, Bettlach, Switzerland). Randomisation was carried out preoperatively after inclusion criteria were met. Closed, identical envelopes were placed in a box and were drawn by an uninvolved nurse. Only patients over the age of 50 with unilateral AO-type C fractures without any other injuries of the upper extremity were included. Patients with intercarpal injuries such as SL-ligament dissociation, fractures older than 8 days, open fractures and patients with premorbid conditions precluding surgical intervention were excluded. Inclusion criteria were deliberately strict to limit this study to fracture patterns not amenable to other means of fixation.

This study was approved by the institutional ethical committee and written consent was obtained from all patients prior to their participation. In sample size determination, based on 0.8 power (p = 0.05, two-sided) to detect a difference of one standard deviation (assumed to be 10°) in motion after 12 months between two balanced groups, 17 cases would be needed. With an assumed follow up rate of 85% 20 patients per group were required.

All patients with radius fractures were followed up after 6 weeks, 3, 6 and 12 month postoperatively. Hardware was removed in all patients after 6 months. Only 42 patients with unilateral AO-type C1, C2 and C3 fractures of the distal radius did the complete follow up of 12 months and thus were included. 8 Patients were lost for follow up, one died due to unrelated cause and 7 could not be reached or were unwilling to return for the final follow up.

Group 1 (the Palmar Plate Group) were treated by fracture fixation with a palmar, plate allowing multidirectional, angle stable screw placement. This group included 22 right-hand dominant patients, 19 women and 3 men, with a mean age of 67.7 (range 52-92) years. The left extremity was injured in 12 patients (all non-dominant) and the right in ten (all dominant). All injuries occurred as a result of a fall onto the hand. Ten fractures were classified as AO-type C1, 7 as AO-type C2 and 5 AO-type C3.

In Group 2 (the Dorsal Plate Group) fracture fixation was performed with the dorsal Pi plate. This group included 20 right-hand dominant patients, 17 women and 3 men, with a mean age of 67.6 (range 52 - 85) years. The right extremity was injured in 8 patients (all dominant) and the left in 12 patients (all non-dominant). Except for one patient who sustained the injury in a motor vehicle accident, all other injuries were caused by falls onto the hand. Nine fractures were classified as AO-type C 1, 5 as AO-type C 2 and 6 as AO-type C 3. Patients underwent open reduction and internal fixation with either a palmar, angle-stable plate (Aptus Radius Plate, Medartis GmbH^®^, Basel, Switzerland) (Volar Plate Group) or a dorsal Pi-plate (AO-ASIF Pi-Plate, Synthes^®^, Bettlach, Switzerland) (Dorsal Plate Group). Surgical techniques and postoperative treatment and assessment have been described in the previous article [5]. All postoperative examinations were performed by a surgeon other than the primary surgeon, but for reasons of patient satisfaction, the primary surgeon saw the patient on every visit as well. In addition to the general assessment, including the Gartland Werley Score, the DASH score was taken 12 months postoperatively to subjectively rate patient activity and satisfaction. Furthermore development of posttraumatic osteoarthritis was evaluated based on the score developed by Knirk and Jupiter [10]. The categoric variables were analysed using SPSS^® ^(SPSS GmbH Software, Munich, Germany, Version 11.5.1) software. After explorative analysis, the Student-*T *test was used except in 2 occasions were the Mann-Whitney test was applied when the Kolmogorov-Smirnov test showed that non-parametric variables were not distributed normally. The level of significance was set at p < 0.05.

## Results

**Figure 1:**
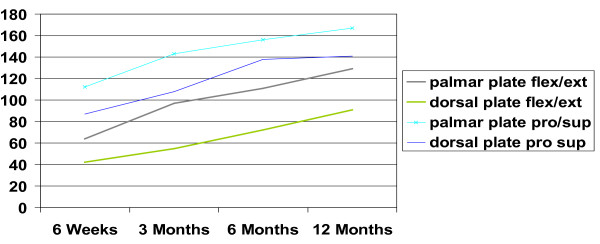
**Development of Pro/Supination and Flexion/Extension**.

**Figure 2:**
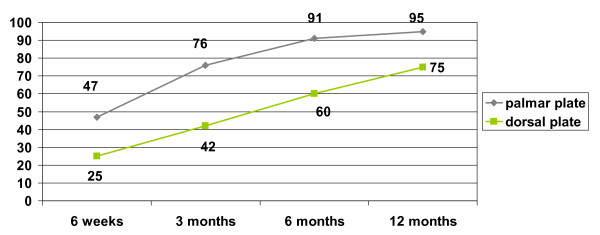
**Gripstrength compared to the uninjured side**.

**Figure 3:**
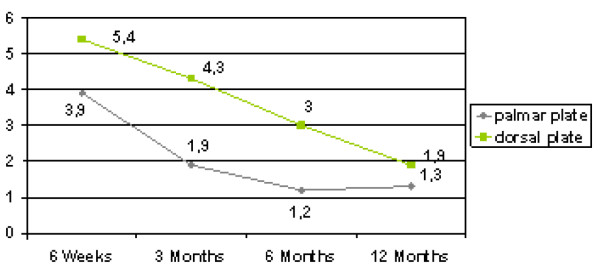
**Pain levels during active motion**.

There was statistically no significant difference between the two patient groups in respect to age, fracture type, hand dominance and gender. The ranges of motion of the wrist and forearm showed statistically significant differences. (Table 1) The palmar plate group showed an increase from 63.6 degrees at 6 weeks for combined flexion and extension to 129 degrees 12 months postoperatively, which was significantly higher than the range in the dorsal plate group, which increased from 42 to 92 degrees 12 months postoperatively (p = 0.007 6 weeks, p < 0.001 (highly significant) at 3, 6 and 12 months). Ranges of motion for radial and ulnar deviation as well as pronation and supination were significantly different between the dorsal and the palmar plate group after 12 months. When range of motion was compared to the non injured hand results of flexion/extension (87 vs. 68%, p = 0.001) and radial/ulnar abduction (93 vs. 78%, p = 0.006) were statistically significantly increased in the palmar group, while no significance was seen in pro-, and supination. (Figure 1) Grip strength, documented as a percentage of the contralateral side, showed significantly better results in the palmar plate group. Over the course of 12 months, grip strength was significantly higher than in the dorsal plate group (p = 0.031 at 6 weeks, p = 0.001 after 1 year).(Figure 2) Postoperative pain assessment revealed no significant differences at rest between the two groups (Mann-Whitney test). However, during active use, pain levels in the dorsal plate group were significantly higher until 6 months postoperatively (p < 0.01), but failed to show a statistical significance after 1 year (p = 0.053) (Figure 3).

**Table 1:** Function

Months	1.5		3		6		12	
Palmar Dorsal	V	D	V	D	V	D	V	D
**Flex/ex**	64 ± 28*	42 ± 16	97 ± 27*	55 ± 23	111 ± 20*	72 ± 16	129 ± 20*	92 ± 33
**Rad/uln**	35 ± 15	28 ± 15	49 ± 10	38 ± 15	57 ± 8	47 ± 10	54 ± 7	47 ± 20
**Pro/sup**	112 ± 42	87 ± 36	143 ± 23*	108 ± 42	156 ± 15*	138 ± 17	167 ± 14*	141 ± 33
**Grip strength**	47 ± 21*	25 ± 16	76 ± 20*	42 ± 18	91 ± 12*	60 ± 18	95 ± 10*	75 ± 21
**Pain**	3.9 ± 2.1*	5.4 ± 1.7	1.9 ± 1.1*	4.3 ± 1.5	1.2 ± 0.6*	3.0 ± 1.6	1.2 ± 0.5	2.0 ± 1.5

*Significance when p < 0.05, Flexion/Extension, Radial/Ulnarduction, Pro/Supination

Posterior-anterior and lateral radiographs were assessed and after 12 months, the radial inclination angle was similar between the palmar plate (24.1 degrees) and the dorsal plate group (22 degrees), with no statistically significant difference between the two groups (p = 0.29) (Table 2). The palmar tilt angle did reveal a significant difference between palmar (9 degrees) and dorsal plate group (13 degrees) (p = 0.024). The evaluation of ulnar variance showed similar results between palmar (0.19 mm) and dorsal plate group (1.32 mm), again with no statistical difference between the two groups (p = 0.068). Carpal sag showed statistical significance between palmar group (1.2 mm) and dorsal group (7.5 mm, p < 0.001).

**Table 2:** Radiological results

Months	1.5		3		6		12	
Palmar Dorsal	V	D	V	D	V	D	V	D
**Palmar tilt**	9 ± 5	15 ± 6*	9 ± 5	14 ± 5*	9 ± 5	13 ± 5*	9 ± 6	13 ± 5*
**Radial inclin**	23 ± 4	21 ± 5	23 ± 4	21 ± 4	23 ± 4	22 ± 4	24 ± 5	22 ± 4
**Rad height**	11 ± 2	11 ± 3	11 ± 2	11 ± 2	11 ± 1	12 ± 2	11 ± 2	12 ± 2
**Ulnar variance**	0.11 ± 1	0.95 ± 3	0.23 ± 1	1.75 ± 3	0.21 ± 1	2.0 ± 3	0.19 ± 1	1.62 ± 3

*Significance when p < 0.05

Osteoarthritis was evenly distributed among both groups with grade one occurring in 7 patients of the palmar group and 8 in the dorsal group, while grade 2 was found in 2 and 3 patients respectively. DASH score was 10.5 in the palmar group and 14.3 in the dorsal group, failing to show statistical significance (p = 0.093, Mann-Whitney Test) The Gartland-Werley score identified statistically significant differences between both groups. Patients in the palmar plate group showed a score of 2.1 compared to 9.2 in the dorsal group (p = 0.001).

No cases of posttraumatic non-union were observed. No case of plate or screw loosening was encountered. Nine complications occurred in six patients in the palmar group. The most common complication was transient paraesthesia in the median nerve in 5 cases which resolved the latest after 6 weeks, not requiring carpal tunnel release. Complex regional pain syndrome Type 1 was diagnosed in two patients. Both improved with physiotherapy and nasally applied calcitonin. Furthermore one tendon irritation, an adhesion of the FPL tendon treated with hardware removal after 4 months occurred. In one patient SL- dissociation was missed intraoperatively and only noticed 6 weeks later. In the dorsal group 11 complications occurred in 7 patients. One patient complained of transient radial nerve dysaesthesia which resolved at the 3 months follow up appointment. Median nerve irritation occurred in one patient, also resolving spontaneously, while CRPS developed in 3 patients and was treated in the same way as in the palmar group. Three secondary fragment dislocations were seen in the dorsal plate group, all in AO-type C3 fractures. Two patients underwent revision, with conversion to a palmar plate, the third refused a second operative intervention. In this patient an intraarticular step of more than 2 mm remained, while another patient of this group developed an ulnar impingement syndrome, requiring ulnar shortening osteotomy. Also two ruptures of the EDC II tendon occurred and were treated with a tendon transfer to EDC III. No complications were seen after hardware removal in both groups.

## Discussion

While AO type A and B fractures have been treated with palmar plates for an extended period of time, highly comminuted fractures have either been treated with a dorsal or combined approach, but not regularly through a palmar approach only. Only the invention of angle stable implants has opened the door to consider this approach in severely comminuted fractures. While palmar fixed angle implants could be the future for treatment of most Colles' fractures, the dorsal approach remains a good choice in highly comminuted fractures with a metaphyseal defect, when a bone graft is also required [2]. A previous study from our workgroup was able to show a faster functional recovery with palmar plates, but it remained unclear if these advantages would persist over an extended period of time [5]. Results show an advantage with the palmar plating system within the 12 month period. Patients regain most function and strength during the first 6 months, afterwards, only a slow, yet still measurable progress can be anticipated [11-13]. Function and strength were significantly improved in the group with palmar plates. This finding was present in flexion/extension, pro-/supination and radial/ulnar abduction. Although dorsal plates did show a comparable increase in range of motion, palmar plates had better function from 6 weeks on and this advantage was not lost over 12 months. Also postoperative grip strength was significantly better at 12 months after use of palmar plates, with similar results to those of other studies using palmar plates [1,2,6,7,11,12,14-17]. Interestingly the early postoperative functional advantage prevailed and thus can be regarded as the cornerstone to successful rehabilitation. This shows that functional recovery with palmar implants is not only faster but also more complete within 12 months. It can be hypothesized that longterm results may be similar as plate removal does improve functional outcome in patients with Pi-plates [2]. Patients with dorsal plates benefited from hardware removal more than patients with palmar plates. They experienced a major reduction of pain levels so that no significant difference between both groups was present after 12 months. Likewise an increase in grip strength after plate removal was seen in the dorsal group. Nevertheless, this group did not completely catch up in terms of strength and function, where statistical significance prevailed after 12 months. Hardware removal cannot be expected to substantially increase ROM or decrease pain levels in patients with palmar plates. The Gartland-Werley score, which incorporates subjective data from patients among objective data, verified a faster recovery in the palmar plate group with significantly lower scores in the palmar plate group. On the other hand, the DASH score, which is subjective and based solely on patient perception, failed to show statistical significance between both groups. This means that patients with dorsal implants adapt to the situation comparable to patients with palmar plates and return to activities of daily living in the same manner.

Radiological results did not show statistically significant difference in regard to radial inclination. Palmar tilt did show a slightly increased result in the dorsal group compared to the palmar group. This proved to be statistically significant. It seems that if increased palmar tilt is desired, some degree of "overcorrection" has to be obtained. This may prove difficult since palmar plates would need additional bending. Nevertheless the palmar tilt of 9 degrees in palmar plates is sufficient in terms of function. A steeper palmar tilt in dorsal plates may be the result of the surgical technique, when the whole distal part of the fracture is reduced to the palmar side with the dorsal plate rigourosly preventing dorsal displacement. This aspect also can be noticed in the carpal sag. The carpal sag was increased in the dorsal plate group, which shows that a complete palmar shift of the fragment and the carpus occurs when the fracture is stabilized from dorsally. This may be due to a straight plate being applied to a curved dorsal aspect of the radius, especially as features like the Lister's tubercle would require a dorsal bend of the plate. A palmar overcorrection will, in some cases, lead to palmar dislocation of the fractures which has occurred in three patients. It has to be stated clearly that, in terms of anatomical restoration, a dorsal implant is by no means inferior to a palmar implant, and in certain aspects such as palmar tilt proved more effective in our group. Nevertheless, radiographs are not the only decisive aspect to judge a plating system as function is the most important aspect for the patient.

The complications encountered in the patients are similar to those described in the current literature and our earlier articles [4,5]. For dorsal implants, complications rates as high as 60% have been described [2]. The most troublesome complication of all, secondary dislocation resulting in a loss of reduction, only occurred with dorsal implants. Especially in AO-type C3 fractures, the lunate facet fragments are very challenging to reduce and retain in position with a dorsal plate only. This may also be due to the design of the Pi-plate, which only offers angle stability when pins are used. In a biomechanical evaluation, Martineau et al. have shown that smooth pegs provide less stability than screws in palmar plates [18]. When dorsal plates are used, the palmar, proximal angulation of the pins and screws will further increase the possibility of dislocation, opposed to the palmar buttressing effect in palmar plates. Because pins are smooth, they do not develop comparable retaining power as screws may let fragments slide palmarly thus causing secondary displacement. Especially in multifragmentary situations it is impossible to secure every fragment with a pin and an additional screw. Palmar buttressing proves advantageous for this problem, by stabilising the fracture from the side with the thicker cortex and avoiding the dorsal comminution zone altogether. Although not present in our patients we are well aware of the fact that some palmarly treated fractures may also show dorsal dislocation and may require dorsal buttressing. With regard to extensor tendon irritations, the second most frequent complication of the dorsal plate, this concurs with a previous study of ours [4]. Despite meticulous development of retinacular flaps to protect the transverse aspect of the plate from irritating extensor tendons, this will not be successful in all cases. Tendon problems cannot be blamed solely on the Pi-Plate, but are due to the presence of any hardware in the dorsal extensor compartments, as shown by tendon ruptures due to protruding screws in palmar plates [19,20]. Therefore no estimation about possible lower complication rates with other dorsal implants that differ in shape and/or diameter can be given although it can be hypothesized that similar complications will be encountered regardless of the shape or material of the implant. It also has to be noticed that no complications were encountered after hardware removal.

There are limitations to this report. The cohort of patients was collected at a tertiary care center with expertise in both plating systems. It is not known whether these results can be generalised, as there is a referral-bias in our patient population. Also our inclusion criteria were deliberately strict to limit our patient population to elderly patients in which plate fixation may be problematic and produce less than ideal results. These patients require stable fixation for poor bone quality to allow quick rehabilitation [7]. This again may increase complication rates compared to other studies when younger patients are included. Another weakness may be the follow-up period of one year. It can be righteously argued that such a period cannot be appropriately regarded as long term. Still, it has been shown by Kreder et al. that one year follow-up is sufficient [20]. Nevertheless our follow up is insufficient to allow conclusions about "true longterm" occurrences such as posttraumatic osteoarthritis which may arise after more than a decade [10].

Overall we have found an advantage of palmar over dorsal plating in comminuted fractures of the distal radius, which supports our earlier findings [5]. While it can be hypothesized that differences in function and radiological outcome will level out over the course of several years, the study has shown the key advantage of the palmar plate to be the faster recovery time. A faster recovery will not only reduce the cost after this injury, but especially in the elderly patient population may restore individual independence thus possibly preventing nursing home placement. While late complications of different plating systems may be similar, it is especially the elderly population who benefits most from faster recovery.

## Competing interests

This study was in part financially supported by IBRA and by the University of Wuerzburg.

None of the authors has any conflict of interest in terms of commercial or financial involvement. No agreement with IBRA was made regarding the prohibition of publishing positive or negative results.

## Authors' contributions

MJ drafted the manuscript, was involved in the design of the study, did the statistical interpretation and analysis

JG carried out the examinations, was involved in the development of the study

RJ developed the design of the study, carried out the examinations

All authors performed the surgeries.

All authors read and approved the final manuscript.
